# Evaluation of an exercise program incorporating an international cycling competition: a multimodal intervention model for physical, psychological, and social wellbeing in residential aged care

**DOI:** 10.1186/s12877-024-05033-x

**Published:** 2024-05-17

**Authors:** Ruth Brookman, Zac Hulm, Leigh Hearn, Joyce Siette, Nitish Mathew, Saili Deodhar, Angela Cass, Jamilla Smith, Belinda Kenny, Karen P. Y. Liu, Celia B. Harris

**Affiliations:** 1 The MARCS Institute for Brain, Behaviour and Development, Western Sydney University, Locked Bag 1797, Penrith, NSW 2751 Australia; 2 Harbison, 2 Charlotte St, Burradoo, NSW 2576 Australia; 3 School of Health Sciences, Western Sydney University, Locked Bag 1797, Penrith, NSW 2751 Australia; 4 Department of Rehabilitation Sciences, The Hong Kong Polytechnic University, Hung Hom, Hong Kong

**Keywords:** Multidimensional, Technology, Exergaming, Virtual experiences, Physical activity, Cycling competition, Older adults, Social networks, Self-efficacy, Depression

## Abstract

**Background:**

The transition into residential aged care is frequently associated with a reduction in physical activity, social engagement, and emotional wellbeing. Our aim was to evaluate the impact of a 26-day international cycling competition (Road Worlds Competition for Seniors), incorporating elements of exercise, audiovisual cycling footage, social engagement, and gamification, on the physical, psychological, and social well-being of aged care residents. We aimed to use findings to inform the development of a multi-modal intervention model to maximise wellbeing for older adults.

**Methods:**

Residents (*N* = 32) participated in a mixed-methods single-group intervention pilot study that compared pre-and post-competition measures for the following wellbeing domains; physical, psychological, and social. In addition, interviews were conducted with residents (*n* = 27) and staff (*n* = 6) to explore their experiences.

**Results:**

Measures identified significant improvements across multiple wellbeing domains, including functional fitness, depression, self-efficacy, and social network sizes. Findings from the interview data indicated that the multimodal components involved in the program delivery were valued by staff and residents who enjoyed the gamification, audiovisual cycling footage, social engagement, opportunities for reminiscence, and camaraderie between peers, staff, and volunteers.

**Conclusions:**

Findings highlight a constellation of benefits across physical, psychological, and social domains of wellbeing and inform a model for innovative multidimensional programs in residential aged care. The benefits for residents with varying physical and cognitive abilities support the use of creative strategies that maximise inclusion and engagement for residents.

**Supplementary Information:**

The online version contains supplementary material available at 10.1186/s12877-024-05033-x.

## Introduction

In residential care, older adults often experience health conditions that impact on multiple areas of wellbeing, including physical mobility [1], independence [2], and access to experiences and social connection [3]. The World Health Organisation (WHO) encourages a holistic approach to supporting healthy ageing by providing activities that address multiple wellbeing domains [4]. Researchers have increasingly focused on multifaceted non-pharmacological interventions that can potentially benefit older people across more than one wellbeing domain including interventions that are meaningful and preserve the identity of older people, and especially those in residential care [5].

### Risks to wellbeing in residential aged care

#### Physical wellbeing

For older people, the transition into residential care is frequently associated with a reduction in physical activity [6]. Independently mobile older adults in residential care spend 85% of their time in sedentary activities, and only 3% in light to moderate intensity physical activities [7]. This is concerning given the numerous benefits of maintaining a physically active lifestyle, such as improvements to quality of life and ageing trajectories. For example, older adults who engage in regular physical activity have reduced risks of physical disease, cognitive decline, and depression (see [8] for a review). However, there are several challenges and risks that restrict the nature of physical exercise in residential care, such as pain exacerbation, fear of falling, and mobility impairments [9]. This highlights a need for motivational strategies and inclusive programs targeted to ranging physical abilities, especially as many residents recognise the role of physical activity in maintaining their mobility, balance, health, and wellbeing [10].

#### Psychological wellbeing

Older adults in residential care are more likely to experience depression than their community-dwelling peers and are at higher risk of suicide [11], with increased risk for those over 85 years whose access to exercise and social interactions is limited [12]. Depression therefore poses a significant risk to residents due to social isolation, chronic health conditions, and limited autonomy. Depression can exacerbate physical health issues, diminish overall quality of life, and increase mortality rates [13]. A comprehensive approach to depression management in residential aged care is imperative and can include person-centred care models that foster identity, self-esteem, social connections and physical activity [14].

#### Social wellbeing

Social networks can have protective role for older adults, as larger network sizes are associated with better quality of life [15] and reduced cognitive decline and risk of developing dementia [16]. In contrast, aged care residents often have few visitors and small social networks [17]. Social isolation is a risk factor that impacts on mental, cognitive and physical wellbeing and mortality [18, 19]. Conversely, poor mobility can pose a further barrier to residents forming new social connections in care [20]. The present study posits that a multi-faceted exercise program that promotes social connection and a sense of community may effectively increase social networks, in addition to improving the physical and psychological wellbeing of residents.

### Program components to promote wellbeing

Due to the need to support older people in residential care across these multiple wellbeing domains, there is impetus to design multi-component interventions that can yield benefits for physical, psychological, and social wellbeing. Prior research suggests several candidates for program components that may have additive benefits when combined into a single intervention, and potential methods of enhancing exercise programs to target psychological and social wellbeing alongside physical wellbeing. In particular, there is evidence to suggest that gamification strategies and technology can be combined with exercise programs to enhance wellbeing across multiple domains in residential aged care settings.

#### Gamification strategies

Research suggests that that gamification strategies—like goal setting, competition, leader boards, and feedback on progress—are more effective in helping older adults adhere to an exercise regime compared to verbal instruction and health education alone [21]. A qualitative study examining the benefits of exergames found that older adults reported an increased sense of competence and enjoyment of the playful competitive atmosphere [22]. It is possible that gamification approaches may promote social networks in residential care settings, as such approaches are known to increase motivation, social engagement and the emotional wellbeing of older adults [23].

#### Exercising and technology

The challenges associated with conducting group exercise program outdoors mean that indoor activity rooms are often the preferred location for exercise in residential care [9]. While activity rooms are safe, they do not allow residents to access the known wellbeing benefits of nature and outdoor settings (e.g., [24]). As an alternative, exergaming (exercising using audiovisual footage of outdoor environments) can be used to create engaging fitness experiences. In this form, users can be fitted with fitness equipment such as treadmills or stationary bikes that sync with audiovisual footage of different locations, allowing users to be transported to and experience a different environment – such as the outdoors, using multisensory audiovisual cues [25]. When this technology is interactive in real time, it is known as virtual reality technology (VRT), with different levels depending on the degree of immersion they facilitate for the user [26, 27]. Following the taxonomy put forward by [26], VRT facilitates a virtual experience across three levels of immersion: Level 1 (non-immersive) employs a desktop computer to navigate a virtual environment; Level 2 (semi-immersive) involves the projection of visual content onto one or more screens, and; Level 3 (fully immersive) uses a head-mounted display of visual content that is worn by users to provide an enveloped digital experience [26]. A recent literature review suggests that non-immersive and fully-immersive VRT are used more frequently than semi-immersive VRT [27]. While it is argued that the contemporary use of the term VRT refers to technology that is interactable in real time [28], in clinical settings, the term VRT is used more broadly and colloquially to include any technology that promotes a virtual experience (e.g., [29]). Therefore, the levels of immersion of VRT (and non-interactive audiovisual technology) are important to consider, as they may have different inherent risks and benefits when combined with exercise, particularly for aged care residents [30].

According to Transformational Learning Theory [31], VRT can provide situational challenges to older adults. Meeting challenges virtually has the potential to shift residents’ beliefs and assumptions regarding their abilities as an older adult in care [32]. In theory, pre-recorded cycling audio video footage (even when the technology is non-interactive) could facilitate a similar experience for older adults and be combined with physical exercise to provide a challenging situation – such as cycling uphill or across rough terrain – with associated improvements to self-perception in real world situations. However, the potential role for appropriate levels of technology to be selected for the aged care setting (including non-interactive audio-visual technology) and combined with exercise programs to additively enhance the psychological and social benefits of exercise has not yet been studied.

The additive benefits of combining physical exercise and technology have, however, been examined in older adults using physical outcome measures, with improvements seen in balance, mobility, and muscle strength (see [33] for a review). A study in aged care found residents cycled for longer and at a higher intensity while watching television [34], and another small study found residents cycled for longer and further when VRT was used, due to the enjoyable and motivating visual input [35] (see also [36]). However, cycling interventions that require sitting and balancing on a stationary bike may exclude a significant proportion of aged care residents due to physical limitations including poor core strength and balance.

### The motiview road worlds competition for seniors

Research dedicated to the evaluation of multimodal physical interventions in residential aged care settings is scarce [37] and has often not identified which program features are beneficial or considered the full constellation of possible benefits across physical, psychological, and social wellbeing. The Road Worlds Competition for Seniors (RWC) is one example of a multimodal program designed for older adults in aged care settings. It is an annual 26-day international competition which incorporates specially adapted exercise bikes with a large digital display, sounds, and ‘real’ time metrics (e.g., distance travelled), that facilitates an international cycling experience. The participant sits on their own chair or wheelchair in front of the cycling apparatus and pedal with their legs or arms while experiencing large-screen video footage featuring a wide range of different global locations, selected from a menu. Competitors can ride in groups, encouraging conversation. The cumulative distance cycled over the month is recorded, and individual and team winners are announced at the completion of the challenge.

### The present study

The aim of this single group mixed methods study was to evaluate the benefits of participation in the 26-day RWC on the physical, psychological, and social wellbeing of aged care residents, and to develop a model for future multi-faceted interventions, highlighting innovative and inclusive aspects that promote wellbeing across domains. As such, pre-and post-competition measures were obtained across physical, psychological, and social dimensions of wellbeing, together with interviews with staff and residents to explore their experiences with the intervention. We predicted that participation in the RWC would be associated with measurable improvements across all three domains of wellbeing.

## Method

### Participants

At the time of the study, there was only one residential aged care organisation in Australia competing in the RWC, so a convenience sample was used. Participation was made available to all residents in this organisation. Residents (*N* = 32) were recruited face-to-face across two locations; Location 1 (*n* = 15) and Location 2 (*n* = 17). The average age of participants (17 Female; 15 Male) was 82.97 years (Range: 70 to 97 years). No residents withdrew from the study. A few residents (*n* = 4) were unable to cycle for the entire month duration due to unrelated health conditions e.g., COVID-19. Residents (*n* = 27) and staff (*n* = 6) also participated in an interview. Medical details such visual impairment, dementia diagnosis, dementia severity and mobility aid use were obtained through chart review and cognitive screening where possible (see Table 1).

**Table 1:** Participant descriptive information (*N* = 32)

Characteristic	N (%)
Gender	
Male	17 (55)
Female	15 (45)
Age (years, mean ± SD)	83.06 ± 7.72
Location	
Location 1	15 (45)
Location 2	17 (55)
Visual Impairment	4 (13)
Dementia Diagnosis	14 (45)
MoCA Categories (*n* = 29)	
No impairment	4 (14)
Mild	13 (45)
Moderate	7 (24)
Severe	5 (17)
Mobility Aide Use	
No aide	16 (50)
Cane	3 (10)
Walker	11 (34)
Wheelchair	1 (3)
Immobile	1 (3)

*MoCA* The Montreal Cognitive Assessment

### Procedure

#### The RWC intervention

##### Equipment and setting

**Fig. 1:**
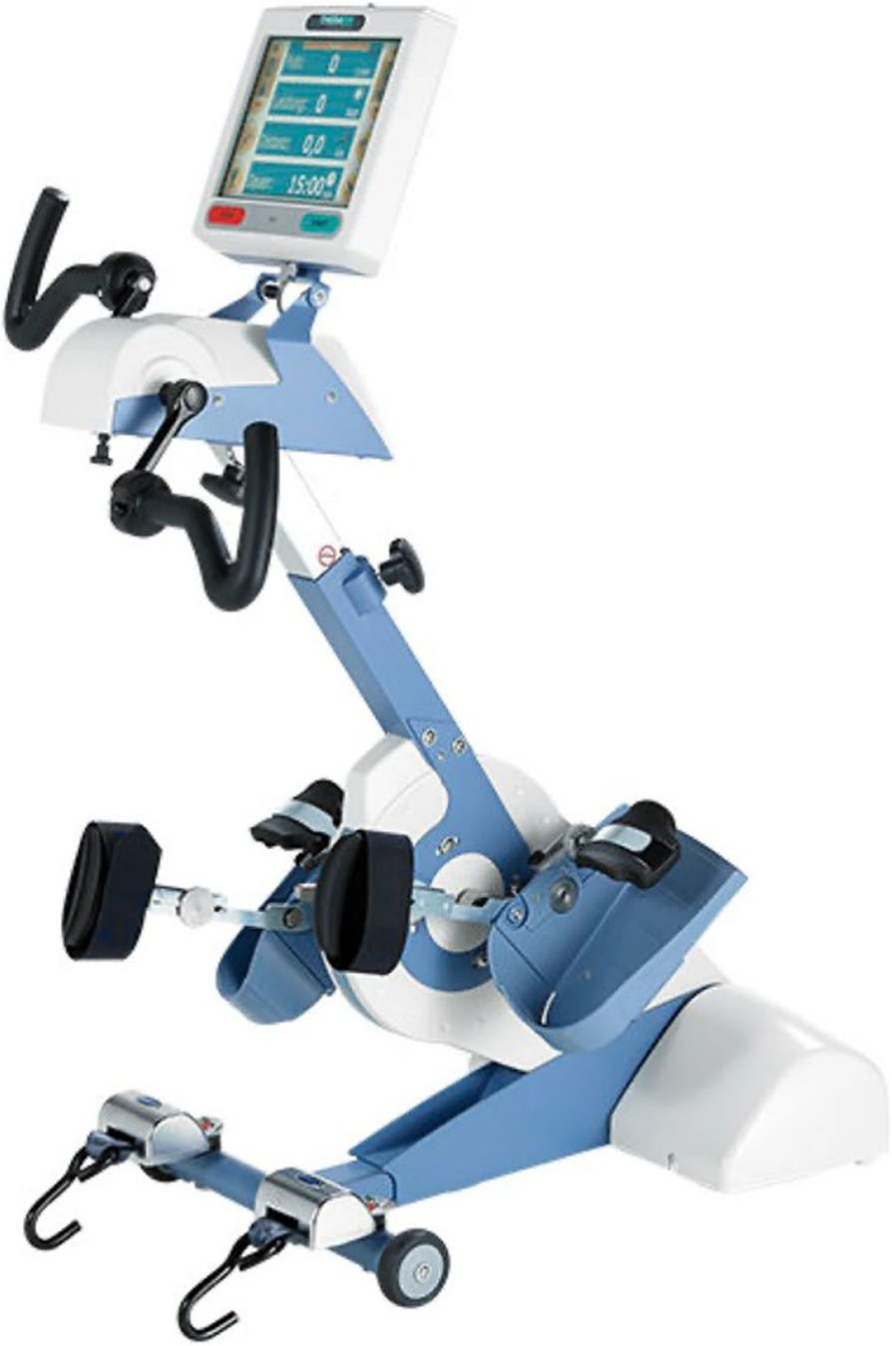
The Thera Trainer adaptive bike [38]

The adaptive Motitech bikes (cycling apparatus) with foot or hand pedal options, enabled participation for those with mobility impairments. Details of the cyclists were added through the Motiview Portal to register an activity, and to enable the generation of reports for each cyclist to track distance travelled, time duration and the resistance level. The small screen on the device also provided cyclists with ‘real time’ metrics such as distance and time travelled during the cycling session (see Fig. 1).

**Fig. 2:**
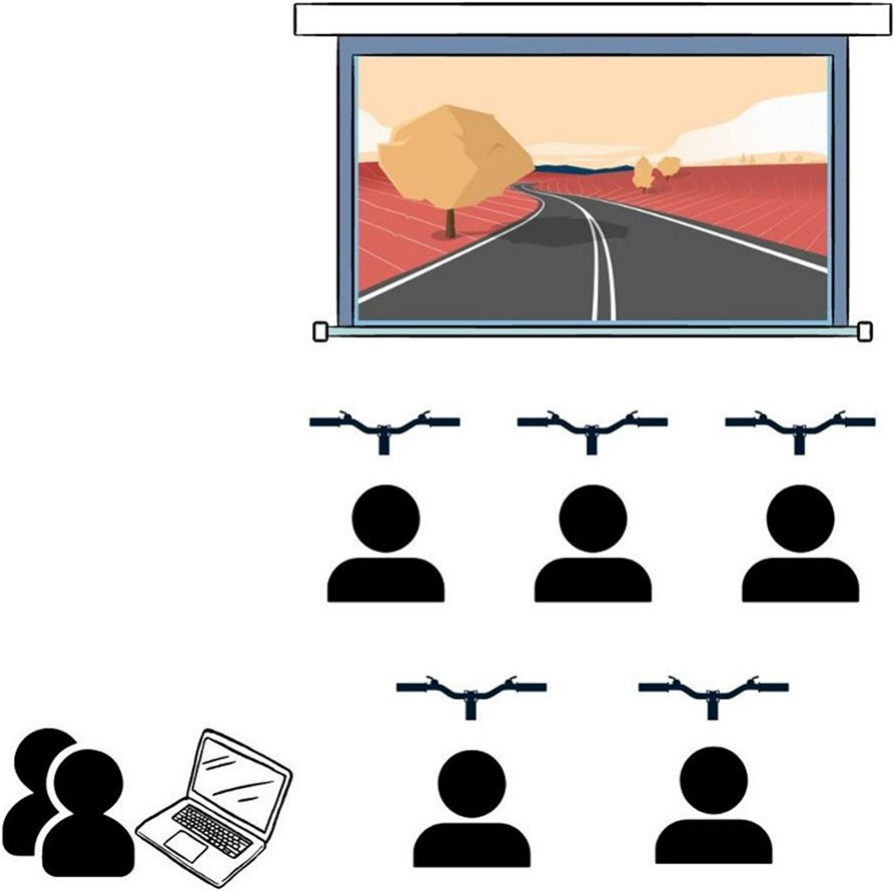
Activity room diagram: Positioning of audiovisual technology and bikes

The RWC was staged onsite in a designated activity area with space for other residents to spectate while waiting to cycle. Each activity room contained 5 adaptive stationary bikes positioned in two rows in front of a large screen. Residents were seated on a chair (or remained in their wheelchair) in front of an available stationary bike. A laptop was used by staff to control the video presentation of a cycling experience on the large screen (see Fig. 2). Residents could cycle during the morning and/or afternoon and they were accompanied by staff to and from the session if they had a cognitive and/or mobility impairment. If residents were present in the competition room at the start of each exercise session, they were invited to choose (via group consensus) one of over 1000 scenic cycling locations including mountainous terrain, countryside locations and bike routes through city streets. If they joined later in the session, they cycled with the existing video. Staff would suggest a location on the occasion/s that residents did not express a preference. The resistance level and duration for each resident was individualised according to personalised goals (distance travelled over the 26-days and length of each cycling session).

##### Gamification strategies

Individual goal setting and achievements were promoted by staff, as residents competed internationally as part of a team. Each day participants had a card placed on their bedroom door with their name and cumulative distance cycled. The cumulative distance travelled over the 26-day competition (recorded by the Motiview system and staff) enabled the identification of winners and place getters. For example, at the end of the competition world champions were chosen in the team, men's and women's categories. The top three in each category received their own certificate and trophy and all participants, regardless of their total distance cycled, received their own medal and certificate. Participants were aware of the competition incentives and the award ceremony following the challenge.

#### Pre- and post-wellbeing assessments

Participants completed wellbeing measures within 2 weeks before and after competing in the RWC, administered by trained research personnel using standardized protocols and instructions.

#### Measures

##### The 5 × Sit to Stand Test (5STS: [39])

The 5STS test measures functional lower limb muscle strength and fitness. Residents were timed while they repeated 5 sit to stand actions. A score of > 16.0 s indicates increased risk of falls, and a small decrease of 2.5 s can provide significant functional improvement [40]. The test was terminated at resident request if they were no longer able to continue.

##### The 2-Minute Walk Test: (2MWT: [41])

The 2MWT is a measure of self-paced walking ability and functional capacity. Residents walked as fast as they could for 2-min, and the distance was recorded. They were able to use their usual walking aid. Further Bohannon and colleagues [42] reported the following mean distances walked by older adults (65 to 85yrs); 144–179 m (males) and 134–155 m (females).

##### The Geriatric Depression Scale-Short Form (GDS-SF: [43])

The GDS-SF is a screening tool design to assess depression in older adults without dementia. Participants responded ‘yes’ or ‘no’ to each item. Scores ranged from 0 to 15 with higher scores indicating a greater number of depressive symptoms. The validity of the GDS-SF decreases when older adults have a cognitive impairment [44].

##### The Cornell Scale of Depression in Dementia (CSDD: [45])

The CSDD was designed to screen for depression in older adults with dementia [46], using a clinician-rated 19-item scale that indexes the following five dimensions: mood and related signs, behaviour disturbance, cyclic function, ideational disturbance, and physical signs of depression. Ratings were provided by aged care staff, with final scores ranging from 0 to 38.

##### The Geriatric Anxiety Inventory (GAI: [47])

The GAI is a 20-item scale designed to assess anxiety in older adults. Participants responded ‘yes’ or ‘no’ to statements read aloud to them about anxiety symptoms over the previous week. The GAI has strong utility in detecting anxiety disorders (e.g., [48]).

##### The General Self Efficacy Scale (GES: [49])

The GES is a 10-item scale designed to measure a person’s self-efficacy. An example question includes, ‘Thanks to my resourcefulness, I can handle unforeseen situations’. The GES is rated on a four-point Likert scale from 1 (‘Not at all true’) to 4 (‘Exactly true’). Total scores range from 10 to 40.

##### Lubben Social Networking Scale (LSNS:[15])

The LSNS is a self-report measure of size of social networks with friends and family and was modified for this study to include measures of friendship (resident and non-resident), staff and volunteers. Higher scores indicate larger and more supportive social networks. The LSNS is a recommended measure for assessing social networks with older adults [50].

#### Interviews

Twenty-seven residents (16 male; 11 female) participated in a post-competition interview (10–15 min) in a quiet room in the residential care facility. Interviews were conducted within 2 weeks of the conclusion of the competition. Health reasons precluded 5 residents from being interviewed. Questions included: ‘What motivated you the most to participate in the RWC?’ and ‘What did you enjoy most (and least) about the RWC?’ Interviews were not audio recorded. Instead, researchers took notes in real-time which included summaries of content and written verbatim quotes where possible. Additional prompts were provided for residents with hearing impairments and/or cognitive impairments associated with dementia.

Six staff members (3 male; 3 female) with roles across leisure (*n* = 2), dementia care (*n* = 2), physiotherapy (*n* = 1) and volunteer (*n* = 1) were interviewed which were audio recorded using a small recording device. Questions included: ‘How do you feel the RWC was an effective activity in promoting (physical/psychological/social) wellbeing?’ and ‘What do you think are the greatest benefits/barriers to RWC participation?’. Researchers took notes in real time, and audio files were transcribed using automated transcript software, Otter.ai. Written transcriptions were listened to alongside the audio recordings and edited for accuracy by two research assistants.

Interviews were conducted by two females; the first author (PhD/M.Clin.Psy) and third author (Occupational Therapy Honours student). Participants were aware of the interviewers’ professional backgrounds and research interests. The first author (registered psychologist) was employed as a research fellow and had extensive field work experience interviewing older adults and caregivers of people with dementia. In addition to undergraduate training in research methods and interviewing, the third author had experience working in health settings.

### Approach to analysis

We compared pre- and post-intervention test scores using paired* t*-test analyses for most measures, using a one-tailed test for statistical significance given the a priori directional predictions [51]. Psychological wellbeing was measured using the GDS or CSDD depending on whether the participant had dementia; scores were analysed together using the non-parametric Wilcoxon Signed Rank pair test.

Interview data were analysed using content analysis by the first and seventh authors (females) using thematic analysis to identify, analyse, and interpret patterns of meaning (see [52]). Both authors had post-graduate training and experience in qualitative research and analysis. The first author had previously met participants during pre-competition wellbeing assessments and was employed as a research fellow in Healthy Ageing: Caregiving and Mental Health. The first author had previous lived experience as a family caregiver of a person with dementia, motivating her interests and may have created bias toward responses from participants with dementia. Participants were unknown to the seventh author, who was employed as a research assistant and collage artist/educator. The seventh author had been employed as a collage artist in residential aged care settings which motivated her research interests and may have biased her toward identifying positive benefits associated with psychosocial interventions. Interview field notes were transferred to an Excel spreadsheet. Written transcripts (staff) and field notes (residents) were compared with each other as themes were identified. Due to visiting restrictions associated with the Covid-19 pandemic, participants did not provide feedback on the data. No software platforms were used to manage the data. Themes relating to the wellbeing domains (physical, psychological, and social) were identified in advance, and remaining themes were derived from the data (see Supplementary Materials, S1, for a completed COREQ 32 [53] checklist).

## Results

### Demographic details

Residents’ medical conditions were obtained through chart review and administration, where possible, of the Montreal Cognitive Assessment (MoCA: [54] (Table 1). The International Classification of Diseases codes were used to classify the sample’s medical conditions, including; neoplasms (*n* = 1); endocrine (*n* = 6); mental/behavioural (*n* = 1); nervous system (*n* = 3), eye (*n* = 5); circulatory (*n* = 21); respiratory (*n* = 4); digestive (*n* = 3); musculoskeletal and connective tissue (*n* = 8); genitourinary (*n* = 4); and external causes (*n* = 1).

### Performance metrics

The total distance travelled during the RWC was 8829 km (*M* = 275 km; *SD* = 286 km), and ranged from 17 to 1035 km. The total duration of cycling during the RWC was 482.30 h (hrs) (*M* = 15.4 h; *SD* = 14.4), and ranged from 0.75 to 45.30 h. These performance metrics were not significantly correlated with demographic variables such as age, gender, dementia diagnosis, and mobility aid use.

### Physical wellbeing outcomes

Paired samples *t*-tests indicated that performance on the Sit-to-Stand-5 test improved from pre- to post-competition. It took residents significantly less time to complete the test after the RWC, *t*(27) = 1.78, *p* = 0.043, yielding a small effect size (*d* = 0.328). Similarly, residents were able to walk further in 2-min post-competition. However, this difference approached but did not reach statistical significance, *t*(27) = 1.50, *p* = 0.071 (see Table 2).

**Table 2:** Pre- and post-competition physical, social, and psychological wellbeing measures

	Pre-test	Post-test				
Outcome variables	*M(SD)*	*M(SD)*	*t*	*df*	*p*	*d*
5-Sit to Stand Test	21.12 (11.19)	17.52 (11.39)	1.784	27	.043*	.328
2-Minute Walk Test	69.61 (27.45)	75.40 (33.93)	-1.500	27	.071	.285
			*Z*		*p*	
Depression			- 2.118		.034*	
			*t*	*df*	*p*	*d*
Anxiety	3.47 (4.08)	2.41 (4.14)	1.601	16	.064	.388
Self-efficacy	32.94 (3.29)	34.65(3.53)	-2.187	16	.022*	-.505
LSNS: Total	7.69 (3.83)	10.77 (4.37)	-4.40	25	< .001***	-.863
Family	1.92 (1.44)	2.23 (1.73)	-1.189	25	.123	-.233
Friends—Residents	1.96 (1.48)	2.38 (1.48)	-2.026	25	.027*	-.397
Friends—Non residents	.54 (.99)	.62 (1.13)	-0.348	25	.386	-.068
Staff	2.22 (1.31)	2.96 (1.48)	-2.642	26	.007**	-.508
Volunteers	.93 (1.21)	2.37 (1.36)	-5.084	26	< .001***	-.978

* *p* < .05, ** *p* < .01, *** *p* < .01; Anxiety = GAI; Self-efficacy = GES; Depression = GDS-SF and CSDD

Interview responses provided by most of the residents supported the physical benefits suggested by the objective measures. Residents reported stronger muscles, increased endurance, feeling fitter, improved circulation, and functional benefits such as being able to walk independently without a mobility aid:

**P6 (Male).** Strengthening my leg muscles.

**P4 (Male).** I can walk! I was having trouble walking to the other side [of the facility] and now I can go on my own! I was at the stage where I nearly needed a walker ... I think it’s [also] helping the blood circulation in my feet.

**P28 (Male).** The fact I was doing exercise was good for me. I had to concentrate on what I was doing for 20 minutes … I had to work hard ... I was getting fitter.

**P2 (Leisure staff).** I [saw] people come from the brink of losing their ability - to be able to move, be able to stand, be able to walk.

**P5 (Dementia care staff).** Walking there, then having a bikes … then walking back. [Prior to the RWC] some people didn't even go for a walk every day. They were just here and this competition … they have to make an effort.

### Psychological wellbeing outcomes

The Wilcoxon Signed-Rank test compared pre- and post-intervention depression measures and show a significant tendency to decrease. Post-intervention scores decreased for 11, increased for 4, and remained the same for 5 participants. Pre-and post-intervention self-efficacy and anxiety measures were compared using a paired samples *t*-tests. Anxiety scores tended to decrease, although this difference was not significant. Results indicated a significant increase in self-efficacy post-intervention, with a medium effect size (see Table 2).

During interviews, residents and staff reported improvements to psychological wellbeing. This was observed ‘in the moment’ as well as over time:

**P9 (Male).** I am happy on the bikes.

**P24 (Male).** Makes your body go and you feel good when you get off the bike.

**P5 (Dementia care staff).** Even if they can't tell you how happy they are [they come back] so easy going, so relaxed, because they did something. And sometimes maybe they forgot what they did, but [they] still have this [positive] feeling.

While the “personal perception that [residents] were unable to undertake exercise” was identified by a staff volunteer as the number one barrier to participation, improvements in self-perception were identified during interviews as a positive psychological outcome. One resident with an amputated leg chose the hand pedal option:

**P25 (Female).** I saw some of the old people riding the bikes ... I didn't think I'd be able to do that … I'd never rode a bike before in my life. The last day I rode ... 50 kms in one go .... staff said to me ‘You got second in the world!’ … Amazing!

**P2 (Leisure staff).** Through constant reassurance and constant encouragement … he's [now] saying … ‘I’m gonna get there.’

### Social wellbeing outcomes

Paired samples *t*-tests indicated that residents reported significantly increased social network size from pre to post competition. Specifically, participants reported increased social networks of resident friends (small effect size), staff (moderate effect size), and volunteers (large effect size). There was no significant change in network size for family and for non-resident friends following the program (see Table 2).

During interviews, volunteers and staff described the importance their role in encouraging and supporting residents to set goals and then work towards them. This finding complements residents’ reports of an increase in their social networks, not only with other residents, but with staff and volunteers.

**P8 (Female).** People say hello to me.

**P2 (Male).** Getting to know the other residents.

**P1 (Volunteer).** It’s about the residents doing the exercise and getting … the mental and physical benefits … getting out of the room and being part of the broader community.

**P2 (Leisure staff).** The volunteers - they do have a great relationship - makes [residents] want to come … [the] level of rapport …spurs them on.

### Opportunities for reminiscence

Interview data indicated that the multimodal activity also appeared to benefit residents in the cognitive domain, as audiovisual cues in the videos elicited memories for residents (e.g., ‘The opportunity for reminiscence’). There was evidence that the experience connected them to memories (e.g., ‘I used to cycle to school’) and identity (e.g., ‘I was a professional cyclist’).

**P5 (Dementia care staff).** Reminiscing ... the volunteers, they're asking, “Where would you like to go today?” “Well, how about London?’. And then the English [resident] is very happy and proud and starts talking about [her homeland].

**P2 (Leisure staff).** A lot of residents have travelled in their lifetime ... [the videos include] places they've been or places they didn't quite get to.

**P1 (Volunteer).** [His] parents were Swiss ... he'd been [there]. And so, you know, he really enjoyed it when we had a couple of Swiss videos.

### Barriers to participation

Staff reported that the competition was generally inclusive of people with dementia, but that suitability ‘depends on the individual’ and required screening of suitability to determine whether the type and severity of dementia could render the experience overstimulating or potentially confusing for some individuals.

**P5 (Dementia care).** You know, you can't generalise … it's suitable for this type of dementia, or it's not. It depends on the individual.

**P2 (Leisure).** Certain things that might be good, like the opportunity for reminiscence and talking about certain things that mean a lot to them. But other forms of dementia that might be a bit more advanced ... it could be a bit of a challenge.

Residents reported that the main barrier to engagement in the activity, was the restricted availability of bikes at popular riding times during the day. As a minor theme, a few residents also mentioned ‘fatigue’ towards the end of the competition.

### Engagement facilitators

#### Motivation is person-centred

When given the opportunity to identify what they enjoyed about RWC, responses included the ‘beautiful’ VR scenery, the competition, and social connection. Each of these program components had the potential to facilitate engagement and enjoyment of the intervention, but to varying degrees depending on the individual. Some residents were motivated by the competition and the opportunity to get fitter, while others were motivated by seeing the movies and others by the social aspects.

**P20 (Male).** The competition was exciting … I love the exercise, excitement, and exhilaration of riding.

**P13 (Male).** I like a challenge, I had none, [and then] I came third in Australia!

**P25 (Female).** The riding; the film clips; you went to different countries.

**P22 (Male).** I didn't get into the competition, but I liked being part of the group.

#### Gamification: a sense of camaraderie

The competition and gamification strategies provided a challenge and purpose for many residents, and tracking their progress served as a tangible measurement of their achievements. Cycling alongside others, while also competing against them, was a motivational factor. Also, competing as a team against other residential aged care facilities created a sense of camaraderie between residents and staff. The formation of a team identity reduced a sense of isolation and enhanced the overall positive experience.

**P5 (Dementia care staff).** They talk to each other. ‘How many [kms] did you do?’, and they were very proud of themselves … I think it's a good motivation. Also, to see … next to your door - the amount of kilometres is good ... every day the number is going up and up.

**P2 (Leisure staff).** I think the camaraderie … they’re riding alongside their friends,

but they're also competing against them … spurring each other on.

#### Social inclusion: A communal experience

Social engagement was facilitated by the adaptability of the Motiview bike that was inclusive of residents with physical impairments ('I'm in my wheelchair and I can do it’) and varying abilities (‘It’s great for all levels of physical fitness and ability’). The set up of the bikes and audiovisual technology appeared ideal for facilitating a communal and social experience. Residents could talk with each other while simultaneously pedalling and engaging with the audiovisual technology. It provided residents with opportunities to socially interact and share new information about themselves, such as previous countries they had visited, and places they wish they had seen.

**P2 (Leisure staff).** Having the four or five bikes by side-by-side … you're there with someone and you get to have a conversation with them about the scenery.

#### Technology that creates a different environment: “It’s the atmosphere”

The large-screen visual display, with video and audio footage moving through beautiful locations worldwide, was also identified by many as impacting positively on residents' mood. The set-up was described by one staff member as “We have a big screen and it's like a cinema, it's the atmosphere”. The visual display with accompanying sound created a sense of anticipation for residents concerning ‘where’ they might be cycling. Residents not only accepted the technology component of the program, but it was a motivation and highlight for them. For example, “Watching the videos” was what they enjoyed most about participating in the competition, as it enabled them to “travel” and “see the world”. That is, although the experience was non-immersive and not interactive, participants described it using language that suggested aspects of a virtual experience.

**P6 (Leisure staff).** They wanted to see new places, [They'd say], ‘Oh, I've seen that one before. I'd like to go somewhere different’. Or they love gardens settings … and places that were really quiet in nature.

**P4 (Male).** I liked the movies, seeing the mountains and the different countries in the world. I felt like I was really there. I haven't seen much of the world; it brings it much closer.

**P3 (Female).** I liked Austria and Switzerland … They were so beautiful!

## Discussion

Findings from this mixed methods pilot study provide a promising evidence base for the use of a multimodal intervention in residential aged care settings. Pre- and post-intervention measures highlighted some significant improvements for residents across physical, psychological, and social domains. The effect sizes for physical wellbeing and social network size of resident friends were relatively small. However, greater effect sizes were evident in increases in self-efficacy (moderate), and social network size for staff (moderate), and volunteers (large). These benefits were supported through analysis of interviews with residents and staff, who cited improvements in fitness and endurance levels, muscles strength, mood, functional abilities and relationships between residents, staff and volunteers. The multimodal components were valued by staff and residents who enjoyed the competition, the technology that supported their cycling experience, and social engagement. Overall, the constellation of benefits seen in our pilot study argues for the continued development and implementation of innovative multimodal programs in residential aged care settings, particularly combining physical exercise with large video displays, gamification, reminiscing, and social interaction, to maximise benefits.

### A multimodal intervention model

**Fig. 3:**
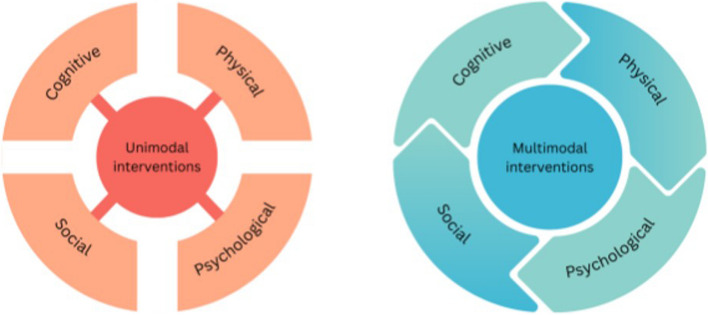
A multimodal model of intervention delivery in residential aged care: The constellation effect on wellbeing

Our research identified the potential benefits of an exercise intervention across multiple wellbeing domains. This “coat tailing” effect suggests a synergistic relationship between wellbeing domains. We therefore propose a multimodal model of intervention delivery in residential aged care settings, as an alternative to conducting a series of interventions targeting single wellbeing domains (see Fig. 3). In the case of the RWC, while the physical domain (cycling) was the focus of the intervention, the multimodal nature of the program – including components such as gamification strategies, large audiovisual displays, metrics in real time, and opportunities for reminiscing and relationship building – contributed to the simultaneous and cumulative benefits across multiple domains. The results from the present study provide a possible framework for the development, implementation, and evaluation of future multimodal interventions.

### Multimodal components impact upon multiple domains of wellbeing

#### Gamification and physical wellbeing

The opportunity to participate in an international cycling competition motivated residents to engage with the physical exercise program. As predicted, residents who participated in the competition made significant gains in their muscle strength and functional fitness, which is consistent with previous research involving a 6-week low to moderate cycling intervention [55]. Although the effect size was small, these gains were made over a one-month period. The competition and gamification components were cited by many as motivating them to achieve more, which may have contributed to greater volumes of exercise (duration and intensity), and greater improvements in physical wellbeing. For example, as competitors, residents were spurring each other on, which corroborates previous research that identified the competition between aged care facilities as motivating [56]. Further, there is evidence that gamification components—such as those adopted by staff (e.g., goal setting, goal tracking, and rewards)—tend to be more effective in helping older adults adhere to an exercise regime rather than instruction and health education alone [21]. Overall, findings suggest that the desire to achieve goals and participate in a competition, is not limited to younger generations but is important across the lifespan and could therefore be harnessed by aged care facilities to engage residents in physical activity.

#### Shifts in self-perception and psychological wellbeing

Bandura (1977) [57] described self-efficacy as a person’s belief about their ability to accomplish tasks and deal successfully with challenges. Gamification components may have contributed to reduced depression and increased self-efficacy observed from pre- to post-competition. Interviews identified that competitiveness and achievement were meaningful for many residents, and research suggests that a sense of meaning is linked with positive benefits for older adults in both physical and psychological domains [58, 59]. Both residents and staff commented on the positive experience of achieving goals. For example, shifts were evident in thought processes from ‘I didn't think I'd be able’ to ‘I came second in the world!’. Bandura proposed that self-efficacy is a dynamic construct that can be enhanced through encouragement from a credible source (e.g., staff), witnessing others (e.g., group activity), and experiencing affective states (e.g., joy). Self-belief influences both the uptake and maintenance of exercise activities in older adults [60]. Findings suggest that the increase in self-belief was not restricted to cycling but generalised to reflect a stronger belief in one’s ability to accomplish tasks. Also, according to Transformational Learning Theory, the use of technology (although non-interactive) that facilitated a virtual experience, may have contributed to the fundamental shift in residents’ self-perceptions of their abilities, by providing a situational challenge – in combination with the physical activity—that were experienced and overcome by residents [32]. The synergistic relationship between the visual and audio display, together with the physical exercise and gamification components of the intervention, may have worked simultaneously to achieve the significant difference in self-efficacy observed in the present study.

Although psychological wellbeing was not targeted by the intervention, residents experienced a measurable reduction in depression. Previous research suggests that social connection protects against depression and physical and cognitive decline for older adults [16]. The interconnection between wellbeing domains suggests that multimodal interventions that address physical and social wellbeing could also reduce depression in older adults [61].

#### Gamification promotes social networks and wellbeing

The gamification component of the RWC also connected some residents with previous achievements and a sense of self. Being part of a team appeared to foster a sense of ‘camaraderie’ or group identity. This is consistent with the research literature suggesting that exergaming interventions (versus regular group training), can improve social connections and quality of life [23]. Incorporating exercise into a group setting yielded increased social networks in addition to improving the physical wellbeing of residents. Interviews highlighted the increased in-the-moment social engagement and relationship building. The group experience involving other residents, volunteers, and staff resulted in significant increases to the size of residents’ social networks within the aged care facility, with important implications for wellbeing [15], although longitudinal studies are required to explore whether these relationships are maintained long-term. The involvement of volunteers to facilitate the RWC was designed to facilitate task completion and safety, but it also enabled an expansion of social networks to include these volunteers as well as enhanced relationships between staff and residents.

#### A shared experience promotes engagement

The staff participants observed that “[residents] were just really keen to see things and pedal away”, which provides an illustration of the way in which the audiovisual component and physical component of the competition worked synergistically to engage and motivate residents. For example, traditional cycling in an exercise room may become monotonous. Although the technology employed was not interactive, residents described the immersive and interactive nature of a virtual experience as the thing they enjoyed most about the activity, adding novelty and excitement to the cycling experience, and providing distraction from the physical exertion of cycling, which can result in more motivation to exercise without perceiving it as strenuous [35]. Also, the “beautiful” scenes from nature have also been associated with positive psychological benefits such as increased calmness, and reduced levels of fear and anger [62]. Therefore, exposure to nature scenes may have contributed to the longer-term benefits to mood seen in the present study.

The residents’ enjoyment of the content in the audiovisual displays, confirms other findings that older adults are open to technology tools for accessing new and stimulating experiences [63]. The technology that supports the experience, had the potential to “transport” residents outside of the confines of their usual environment, connecting residents to outside places, in a similar way to windows connecting older adults with the outside world [64]. In that sense, the non-immersive and non-interactive technology employed in this intervention was still experienced as immersive and virtual by some residents (‘I felt like I was really there’), despite being a relatively low-tech option using a large visual display screen with accompanying soundscape. As such, it enabled those with limited mobility to ‘re-visit’ familiar places and ‘travel’ to places previously unseen.

#### Video footage provides opportunities for reminiscing

Reminiscence therapy employs tangible triggers such as music, photos, and videos, and the multisensory stimulation of technology can help older adults to connect to autobiographical memories of people, places, and events [25, 65]. During the RWC, reminiscing occurred incidentally at times, as staff observed the audiovisual content on the display screens connected residents to their sense of self and to others, consistent with other research on virtual visits to familiar places [66]. Previous research suggests that recalling autobiographical memories can connect people to their identity and promote social bonding (e.g., [67]) and social engagement through storytelling and sharing memories with peers and caregivers [68]. Therefore, the opportunity to reminisce during the competition may have contributed to the benefits to psychological wellbeing in the present study. Future research could follow this up more directly and include measures that test for cognitive and memory benefits.

### A supportive environment matters

Implementing a multimodal program facilitates person-centred care by enabling staff to motivate and engage residents in diverse ways, depending on their individual preferences. Staff drew on the gamification strategies to motivate residents who valued the competition, the virtual “travel” for those residents who like the audiovisual content on the display screens, or the opportunity for social interaction for those who valued it. The role of the residential aged care facility, therefore, is another important component to the interventions’ outcomes. Barriers to participation were addressed by staff at individual and site levels, to encourage participation of residents across a broad range of backgrounds, ages, and abilities. A volunteer program was implemented to ensure the cycling sessions were supervised and residents supported. In addition, individualised goal setting and the recognition of achievements (verbally and through award ceremonies) may have enhanced psychological outcomes and sense of belonging.

### The role of inclusive technology and adaptive equipment

The specific increase to the network sizes of staff, volunteers, and other residents, also suggests that the adaptive features of the Motiview System were effective in addressing some barriers to forming social connection within the residential aged care setting, such as chronic health conditions and mobility problems [20]. Participants could cycle from their wheelchair, or could hand-pedal, promoting inclusion for those who may not benefit from other exercise programs. Residential care facilities tend to use technology that supports a virtual experiences in one-on-one reminiscing contexts [69], especially when it is fully immersive headset VR. However, in the present program, the group-based use of technology to present video footage on a large, shared screen facilitated social interactions, because it was a common experience shared by everyone in the room and included those with lesser verbal ability. The fact that the technology was *not* fully immersive or interactive in ‘real time’, also promoted inclusion for those with dementia for whom full immersion may have been confusing or distressing. For example, staff felt that the severity of dementia could render the experience overstimulating for some individuals, so safety should be assessed on an individual basis.

### Limitations and future directions

Our pilot study is the first to examine the effectiveness of the RWC program. Because only one organisation participated in Australia, the use of a convenience sample was necessary, and potentially limits the degree to which findings can be generalised to other populations. The sample group included residents with a range of co-morbidities, physical and cognitive capacities, and levels of engagement with the activity, which reflects the status of many older adults in residential settings. While our findings demonstrate a variety of benefits across multiple domains of wellbeing, caution should be exercised in the interpretation of our findings, as multiple distinct t-tests are prone to Type 1 errors and make it difficult to control for covariates, especially considering the diversity of our participants and large variation at baseline. While our findings provide a promising foundation for the benefits of multimodal interventions, they should not be overstated. Instead, future research should build on these findings with a larger sample size, and a randomised control trial (RCT), especially to separately test the impact of the different components. Also, further study could explore the effects of various types of technology on benefits, comparing low-tech non-immersive to fully immersive VRT options. Future research could also examine other aspects of physical wellbeing, such as cardio-pulmonary endurance, and whether the increase in social network sizes was sustained beyond the competition. Finally, further research could explore to what extent the different gamification strategies separately or together contributed to general self-efficacy, focusing on delineating the mechanism underlying this benefit for precisely, as well as examining how increased self-efficacy may in turn impact on other domains of wellbeing such as the perceived ability to perform activities of daily living.

## Conclusion

The outcomes of this pilot mixed-methods investigation establish a solid foundation for endorsing the development of multi-component in residential aged care facilities, combining exercise with gamification and goal setting, and audiovisual cycling footage that promoted a virtual experience for some residents and meaningful social interaction for others. Integrating these features, we found initial evidence for positive outcomes across multiple domains of wellbeing, including physical, psychological, and social domains. However, to build on this work and increase confidence in the findings, future research should consider RCTs across multiple sites. The collective advantages observed in the present study underscore the need for the continued development and implementation of innovative multimodal programs within residential aged care environments. As such, a multimodal model of intervention in residential aged care settings is proposed, to highlight the synergistic relationship between wellbeing domains. Multimodal programs that are thoughtfully designed and creatively implemented are therefore likely to maximise benefits and enhance the overall enjoyment and wellbeing of aged care residents.

### Supplementary Information

Supplementary Material 1.

## Data Availability

The dataset supporting the conclusions of this article is available in the OSF repository, osf.io/sx62n.
