# Supplementary material for: Evaluation of an exercise program incorporating an international cycling competition: a multimodal intervention model for physical, psychological, and social wellbeing in residential aged care
Source: BMC Geriatr. 2024 May 17;24:435. doi: 10.1186/s12877-024-05033-x (PMC11100139; doi:10.1186/s12877-024-05033-x)
Supplement: Supplementary file 1 — Supplementary Material 1. [file 12877_2024_5033_MOESM1_ESM.docx]

**Supplementary materials**

S1. Consolidated criteria for reporting qualitative studies (COREQ): 32-item checklist.

| Numbers | Item | Guide questions/description | Response/page |
| --- | --- | --- | --- |
| Domain 1 | | | |
| Research team characteristics: | | | |
| 1. | Interviewer | Authors conducting the interviews? | 1^st^ and 3^rd^ |
| 2. | Credentials | Researchers’ credentials? | p. 12 & 13 |
| 3. | Occupation | Researchers’ occupations? | p. 12 & 13 |
| 4. | Gender | Researchers’ gender? | Female, p. 12 |
| 5. | Experience | Researchers’ training/experience? | p.12 & 13 |
| Relationship with participants: | | | |
| 6. | Relationship | Was there a prior relationship? | No |
| 7. | Participant knowledge | What did the participants know about the researcher? | p. 12 &13 |
| 8. | Interviewer characteristics | What characteristics were reported about the interviewer? | p. 12 &13 |
| Domain 2 |  |  |  |
| Theoretical framework: | | | |
| 9. | Methodological orientation/theory | What methodological orientation was stated to underpin the study? | p. 13 |
| Participant selection: | | | |
| 10. | Sampling | How were participants selected? | p. 7 |
| 11. | Approach | How were participants approached? | In person, p. 7 |
| 12. | Sample size | How many participants in the study? | N=32 & 6 staff |
| 13. | Nonparticipation | How many participants withdrew? | Zero |
| Setting: |  |  |  |
| 14. | Setting | Where was the data collected? | p. 8 & 9 |
| 15. | Non-participants | Was anyone else present? | No |
| 16. | Sample description | What are the sample characteristics? | p. 7 and 14 |
| Data collection: | | | |
| 17. | Interview guide | Were questions provided by authors?  Was it pilot tested? | p. 12  No pilot testing. |
| 18. | Interviews | Were repeat interviews carried out? | No |
| 19. | Recording | Were audio recordings used? | Yes (staff only) |
| 20. | Field notes | Were field notes made? | Yes, p. 13 |
| 21. | Duration | What was the interview duration? | 10-15 minutes |
| 22. | Data saturation | Was data saturation discussed? | No |
| 23. | Transcripts returned | Were transcripts returned to participants for comment? | No. Explanation provided, p. 13 |
| Domain 3 |  |  |  |
| Data analysis: | | | |
| 24. | Number of coders | How many data coders? | Two |
| 25. | Coding tree | A description of the coding tree? | No coding tree. |
| 26. | Derivation of themes | Were themes identified in advance  or derived from the data? | Both |
| 27. | Software | Software for data management? | NA |
| 28. | Participant checking | Did participants provide feedback on the findings? | No |
| Reporting: | | | |
| 29. | Quotations | Quotations given and identified? | Yes, p. 16-21 |
| 30. | Data and findings consistent | Was there consistency between the data presented and the findings? | Yes, p. 15-21 |
| 31. | Major themes | Major themes clearly presented? | Yes, p. 16-21 |
| 32. | Minor themes | Discussion of minor themes? | Yes, p. 18 |
